# The effectiveness of decision aids for pregnancy related decision-making in women with pre-pregnancy morbidity; systematic review and meta-analysis

**DOI:** 10.1186/s12884-022-04402-x

**Published:** 2022-01-29

**Authors:** Rebecca Whybrow, Louise M. Webster, Paul T. Seed, Jane Sandall, Lucy C. Chappell

**Affiliations:** Department of Women and Children’s Health, King’s College London, Westminster Bridge Road, London, UK

**Keywords:** Decision aid, Shared decision-making, Informed decision, Obstetric medicine, Pre-existing medical condition, Previous caesarean section, Mental health, Epilepsy, Rheumatoid arthritis, Multiple sclerosis

## Abstract

**Introduction:**

Women with pre-existing morbidity arising from medical conditions or previous caesarean section are at higher risk of adverse pregnancy outcomes compared to women without such morbidity. Women often face complex pregnancy-related decision-making that may be characterized by conflicting maternal and perinatal priorities. The aim of this systematic review and meta-analysis was to assess randomised controlled trials of decision aids to evaluate whether they are effective at reducing decisional conflict scores and to evaluate what type of decision aids are most effective for women with pre-existing morbidity in pregnancy.

**Methods:**

We searched Medline (via Ovid), Embase (via Ovid), CINAHL (via EBSCO) from the earliest entries until September 2021. We selected randomised controlled trials comparing patient decision aids for women with pre-existing morbidity with usual clinical practice or a control intervention. Study characteristics and Jadad risk of bias was recorded. Meta-analysis by pre-existing morbidity type was performed using Stata 17 and the data was presented with a Forest Plot. Random effects models were used to calculate summary estimates if there was substantial clinical or statistical heterogeneity and post mean DCS scores were described in a sensitivity analysis and presented as a line graph, to improve clinical interpretation of results.. A narrative synthesis of the selected studies evaluated what type of decision aid works and for in what circumstances.

**Results:**

Ten randomised controlled trials, which reported data from 4028 women, were included. Patient decision aids were evaluated in women with pre-existing morbidity who were undertaking pregnancy-related decision-making. Patient decision aids reduced decisional conflict scale scores by an additional − 3.7, 95% Confidence Interval − 5.9% to − 1.6%) compared to the control group. Women with pre-existing medical conditions were more conflicted at baseline and had greater reductions in decisional conflict scale score (mean difference vs. control group: − 6.6%; 95% CI − 9.8% to − 3.3%), in contrast to those with previous caesarean section (mean difference − 2.4%; 95% CI − 4.8% to − 0.1%). There was limited evidence on the effect of decision aids on health outcomes. Decision aids reduced unwanted variation in decision-making support across maternity settings.

**Conclusion:**

Patient decision aids are effective tools to support personalised care planning and informed decision-making in women with pre-existing morbidity. Women with pre-existing medical morbidity were more conflicted at baseline and were more likely to benefit from decision aids. Adoption of aids in this population may lead to improve adherence and health outcomes, warranting further research.

**Supplementary Information:**

The online version contains supplementary material available at 10.1186/s12884-022-04402-x.

## Background

Personalised care is one of the principal objectives of maternity care in the United Kingdom, and internationally. Care centred on the woman, based around her needs and decisions, and where she has genuine choice, informed by unbiased information, should be provided to women with pre-existing morbidities and those without [1]. Women entering pregnancy with pre-existing morbidity arising from medical conditions or previous surgery (such as Caesarean section) are at higher risk of adverse pregnancy outcomes compared to women without such morbidity [2]. These women often face complex pregnancy-related decision-making that may be characterized by conflicting maternal and perinatal priorities. Women may be presented with pregnancy-related decisions where there remains substantial uncertainty due to an absence of research [3, 4]. Additionally, women are likely to have unique experience and knowledge of their condition that will influence their decisions. Decision-making is therefore likely to be distinctive to women entering pregnancy with pre-existing morbidity arising from medical conditions or previous surgery; and potentially characterised by substantial internal conflict.

Shared decision-making is a model of care where clinicians and patients share the best available evidence when faced with the task of making decisions; and where individuals are supported to consider options, to achieve informed preferences [5]. In pregnancy, this is particularly well suited to situations where medication or surgical decisions are to be made, as they require professionals to share information, but importantly where the best course of action may include some uncertainty [4]. Women with pre-existing medical morbidity often have experience in managing their condition and bring important perspectives to the decision-making process. Implementing patient decision aids that have been informed by the ‘International Patient Decision Aid Standards’ (IPDA) [6] is an effective method to improve shared decision-making in different healthcare settings [5]. They are designed to help support patients to make decisions regarding the balance of benefits and risks of treatment choices, and help support patients to synthesise and express their values, opinions and preferences in relation to the treatment decisions [6, 7]. Increasingly, patient decision aids are being introduced into a wide range of maternity contexts to help support clinicians to provide personalised care and to enable women to actively participate in decision-making regarding pregnancy and birth [8].

A recent review of patient decision aids in a wide range of clinical scenarios across obstetrics and gynaecology has identified that aids are useful in reducing decisional conflict scores (DCS) in women [8]. However, it is uncertain whether decision aids are effective in reducing decisional conflict in women with pre-pregnancy morbidity as these studies were not included in this recent review. Such women often face complex pregnancy-related decision-making that may be characterized by conflicting maternal and perinatal priorities and a lack of evidence on which to base decisions [3, 4]. Furthermore, it is not known what types of decisions aids are effective for decision-making in women with pre-existing morbidity.

The aim of this systematic review and meta-analysis was to establish in women with pre-existing medical and surgical morbidity affecting pregnancy whether patient decision aids are effective at reducing decisional conflict scores, improving knowledge and health outcomes. The aim of the narrative synthesis was to evaluate what type of decision aids are most effective in what circumstances.

## Methods

### Protocol development

The study protocol for this systematic review was developed in line with the PRISMA-2020 checklist and registered on the PROSPERO database (http://www.crd.york.ac.uk/PROSPERO/ reference number (CRD42018109005). No ethical approval was required.

### Electronic database and search strategy

A comprehensive search using Medline (via Ovid), Embase (via Ovid), CINAHL (via EBSCO) from Medline 1946, Embase 1947 and CINHAL 1956 until 07/09/2021 was performed. Search strategies were adapted to each database. Searches of exploded title, abstract and keywords “Pregnancy” or “Prenatal Diagnosis,” or “Partuition” (Medline and CINAHL) or “Birth” (Embase) were combined with “Decision Support Techniques” (Medline and CINAHL) or “Decisions Support Systems” (Embase) or “Decision Making”. The randomised controlled trial filter was then applied to the search results.”. The Cochrane Trials Register was accessed via Google Chrome and searched using title, abstract and keywords “Pregnancy” and “Decision support”. Medline and Embase searches were performed individually and then combined in a single Ovid database which was automatically deduplicated. Integration and deduplication of the combined search with the search results from CINHAL and The Cochrane Trials Register was done by hand in Microsoft Word. No unpublished studies were identified by searching for trials registered on https://clinicaltrials.gov/or ISRCTN (www.isrctn.com) and reviewing thesis titles from https://www.worldcat.org/.. References of studies that underwent full text review and relevant review articles were also searched using the snowballing approach. No language restrictions were applied. The study protocol (including the literature search strategy) is detailed in supplementary file 1.

### Study selection criteria

Randomised controlled trials of patient decision aids for women with pre-existing medical or surgical conditions undertaking pregnancy related decision-making was the focus of the systematic review with meta-analysis. Only patient decision aids that were developed with reference to internationally agreed-on criteria that includes information regarding the health condition; the interventions available and the evidence base for them; the possible benefits and harms; probabilities and uncertainties; and the provision of a method for clarifying and communicating the patient’s values were included [6]. Beyond the restrictions listed in the inclusion and exclusion criteria table (Table 1), no other constraints were applied to the study search.

**Table 1:** Systematic review with met-analysis inclusion and exclusion criteria

Inclusion criteria:	Exclusion criteria
**Population** •-Women making pregnancy related decisions in relation to pre-existing morbidity •-Pregnant women with a pre-existing medical condition •-Women with a medical condition who are planning pregnancy •-Pregnant women with a pre-existing surgery pertinent to pregnancy and birth planning •-Women who are planning pregnancy with pre-existing surgery pertinent to pregnancy and birth planning	Any study designs other than RCT
**Design** Randomised controlled trial in which patient decision aids were compared to usual care with or without an information brochure	Any decision aids that do not meet the International Patient Decision Aid Standards (IPDAS)
**Reporting** Trials that reported pre-defined outcomes	

### Data extraction and quality assessment

The titles, abstracts and selected full texts generated from the literature search were independently screened by authors R.J.W. and L.M.W. Data from the trials that met all inclusion criteria were manually extracted and entered a standard extraction table independently from full texts by R.J.W. and L.M.W. The authors were not masked to the results of the study or authors. Where two articles published results from the same study, individual pertinent outcomes were extracted from both articles without repetition of data extraction. The viewers independently assessed each trial’s methodologic quality using the Jadad criteria, a standardized tool that assesses quality and risk of bias of randomized trials [9]. The criteria employ five questions pertaining to randomization, blinding, and reporting of participant withdrawals. Studies are given a score out of five, with higher scores indicating higher quality [9].

### Statistical methods

Decisional conflict was chosen as the primary outcome as it is a patient-oriented indicator of the decision-making process. Decisional conflict is measured using the validated decisional conflict scale (DCS) [10]. The scale relates to women’s uncertainty, how informed they are, the ability to clarify their values and how well supported they are in relation to the decision. The scale is usually used to generate a pre and post DCS percentage score; scores less than 25% are associated with implementing informed decisions, scores above 37.5% are associated with decision delay. Any reduction in DCS mean difference can be considered important as it may move an individual below the threshold to implement a decision (25%); a reduction of more than 12.5% from a baseline of above 37.5% has been validated as clinically effective [10]. Where scale data was presented, the scores have been recalculated to generate percentage scores (x-1)*25 as described in the Ottawa decisional conflict handbook (supplementary file 2). In each trial, individual patient DCS were combined to give mean pre and post DCS for the control arm and intervention arm.

Results were grouped and meta-analysed by pre-existing morbidity type (medical or surgical) using Stata 17 and the data was presented with a Forest Plot. Egger’s test for publication bias was performed using Stata 17 and analysed by pre-existing morbidity type (medical or surgical). Random effects models were used to calculate summary estimates if there was substantial clinical or statistical heterogeneity, as is recommended in the Cochrane Handbook [11]. Where it was not possible to obtain missing data, only the available published data was analysed. If trials reported enough detail on group means and provided no information on associated standard deviation (SD), the outcome was assumed to have an SD equal to another study using the same scale within the same analysis. Mean differences and SD were calculated from 95% CIs or odds ratios, as appropriate. Furthermore, the pre and post mean DCS scores of individual studies were described in a sensitivity analysis and presented as a line graph, to improve clinical interpretation of results. Where there was an absence of DCS baseline data the study was excluded from this sub-analysis. Where there was a three-arm trial the decision aid arm was compared to the usual care arm of the trial and the participant numbers adjusted accordingly.

A narrative synthesis approach [12] that describes the effect of decision aids on women’s knowledge was adopted due to the heterogeneity of the knowledge measurement scales [13]. Furthermore, a narrative synthesis which included an investigation of the similarities and the differences between the health outcomes of different studies was performed, with sub-group analysis in the pre-existing medical and surgical condition groups. In addition to describing the potential effect of decision aids on decisional conflict, knowledge, and health outcomes the systematic review sought to describe what type of decision aid works and in what circumstances do they work. A narrative synthesis of paper, web-based and personalised aids, along with synthesis of information brochures and decision aids was performed. Further analysis of the use of decision aids in different healthcare settings has been performed, with decisional conflict score as the primary measure.

## Results

**Fig. 1:**
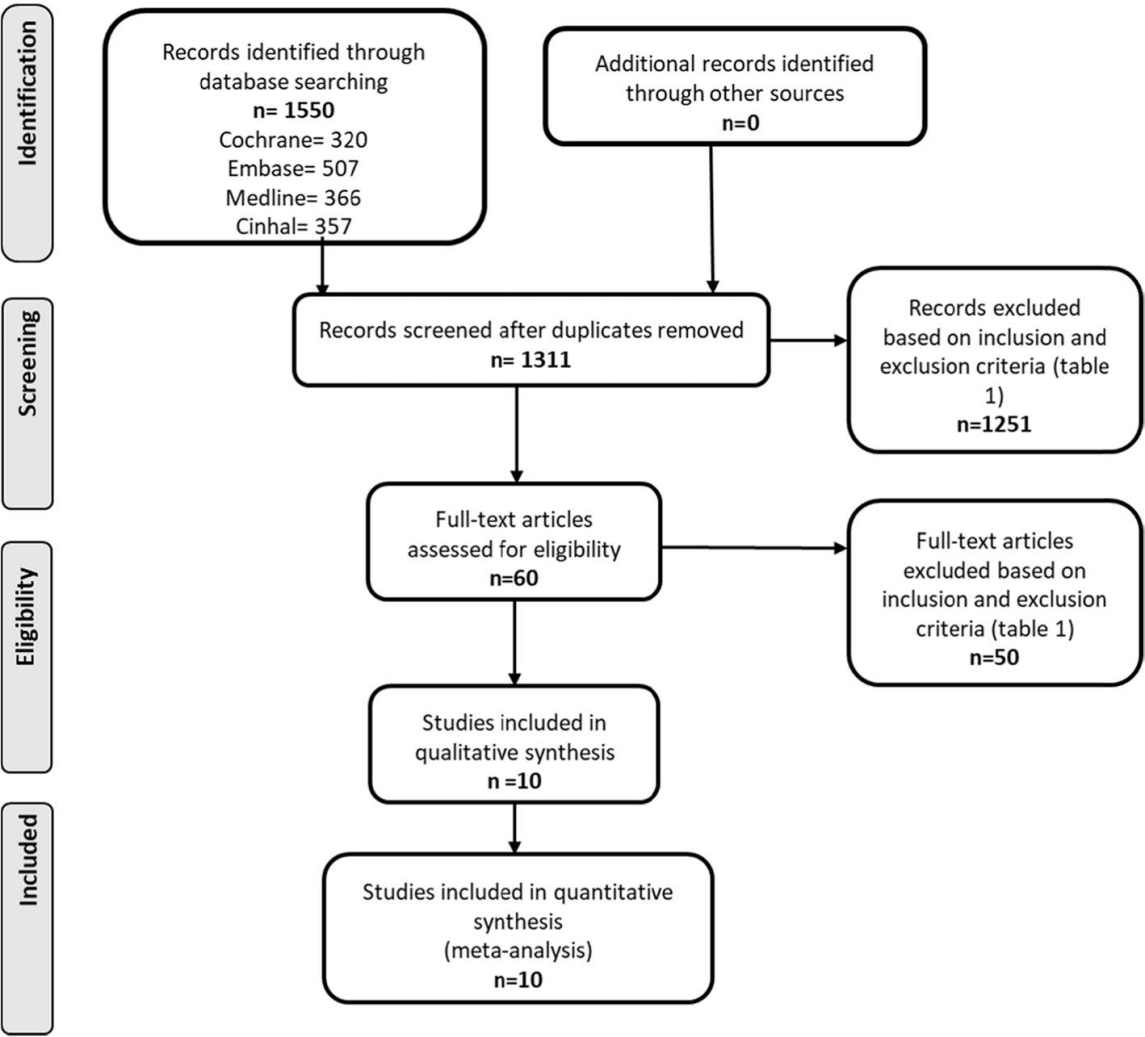
Flow chart reporting identification of randomised controlled trials included for systematic review

Titles and abstracts of 1311 papers were screened, and 60 relevant studies were selected for full manuscript review (Fig. 1). Ten randomised controlled trials that met all the criteria for inclusion were identified for analysis (Table 2) [14–23]. The studies that appeared to meet the inclusion criteria but were subsequently excluded were done so on the basis of the intervention not meeting the IPDAS decision aid standards, a non-randomised methodological approach to testing having been adopted or the women recruited to the study not having a pre-existing medical or surgical condition affecting pregnancy decision-making. A total of 4028 participants were included across the ten trials; the studies were carried out in Australia, Canada, New Zealand, United States of America, and United Kingdom and were published between 2005 and 2020 (Table 2). Seven studies were randomised controlled trials, [14–16, 19, 21–23] two were pilot randomised controlled trials with feasibility and acceptability as primary outcomes [17, 18] and one study was a three-arm comparative randomised controlled trial [20]. Five of the trials included women with a variety of pre-existing medical conditions who were making decisions about treatment and management of their condition in relation to pregnancy [14–18] and five included women with a previous caesarean section who were making decisions about mode of birth [19–23]. The type of intervention varied across the trials, with five of the studies using paper-based decision-aids [14–16, 19, 22] and five a computerised decision aid [17, 18, 20, 21, 23]. Two of the studies included an element of personalisation that included user-specific risk information using a validated prediction calculator that incorporates patient characteristics known during early prenatal care [23] and an interactive deliberation component [21]. The control arms of the randomised controlled trials included usual care, [14–16, 19] specialist services for all or a proportion of women in the studies [17, 18, 20] [22, 23] and usual care alongside an information brochures [21]. The three-arm trial purported to compared two different decision-aids to usual care but the description of the online information arm did not include ‘methods for clarifying and expressing patient values’ nor did it describe providing ‘structured guidance in deliberation and communication’; it was therefore not possible to classify this arm of the study as a decision aid that met the IPDAs criteria and was classified as an online information brochure.

**Table 2:** Randomised controlled trials included in the systematic review and meta-analysis

Study	Population	Intervention	Comparison	Outcome measures	Setting	Timing	Decisional conflict mean difference score (95% CI)	Change in women’s knowledge
**Pre-existing medical conditions**								
Prunty (2008) [14] RCT	Pre-pregnancy women with multiple sclerosis	Paper decision aid booklet IPDAS compliant	Usual care physician care	Primary end: knowledge, DCS Secondary: self-efficacy, certainty, value congruence, bias.	Australian health system. Aid delivered at home or by researcher, not as part of usual care.	Pre-survey - anytime pre-pregnancy. Post survey within 3 weeks.	−5.50 (−10.54, −0.46)	Decision aid increased women’s knowledge
Meade et al. 2015 [15] RCT	Pre-pregnancy and pregnant women with rheumatoid arthritis	Paper decision aid booklet IPDAS compliant	Usual care physician and antenatal care	Primary: Knowledge and DCS; Secondary: Self efficacy, depression, and anxiety.	Australian health system. Aid delivered at home or by researcher, not as part of usual care.	Pre-survey -anytime pre-pregnancy or during pregnancy. Post survey within 12 weeks.	−6.66 (−13.28, − 0.04)	Decision aid increased women’s knowledge
McGrath et al. 2017 [16] RCT	Pre-pregnancy women with epilepsy	Paper decision aid booklet IPDAS compliant	Usual care physician care	Primary: knowledge and DCS Secondary: self-efficacy, certainty, value congruence, bias.	Australian health system. Aid delivered at home or by researcher, not as part of usual care.	Pre-survey - anytime pre-pregnancy. Post survey within 3 weeks.	−10.98 (−21.78, − 0.18)	Decision aid increased women’s knowledge
Vigod et al. 2019 Pilot [17] RCT	Pre-pregnancy and pregnant women with depression	Computerised decision aid IPDAS compliant	Usual care from specialist perinatal mental health care and non-specialist antenatal care plus online information sheet	Primary: Acceptability; Secondary: DCS, PND and Anxiety	Canadian health system. Aid delivered at home or by researcher, not as part of usual care.	Pre survey – anytime. Post survey within 4 weeks.	−7.50 (− 15.23, 0.23)	Decision aid increased women’s knowledge
Khalifeh et al. 2019 Pilot [18] RCT	Pre-pregnancy and pregnant women with depression	Computerised decision aid IPDAS compliant	Usual care from specialist perinatal mental health care and non-specialist antenatal care plus online information sheet	DCS	United Kingdom health system. Aid delivered at home or by researcher, not as part of usual care.	Pre survey – anytime. Post survey within 4 weeks.	−5.30 (− 14.95, 4.35)	No improvement detected
**Pre-existing surgical conditions**								
Shorten et al. 2005 [19] RCT	Pregnant women with one previous caesarean section	Paper decision aid booklet IPDAS compliant	Usual routine antenatal care.	Primary: DC Sand knowledge; Secondary: congruity of decision and outcome.	Australian private obstetric practice. Aid delivered at home or by researcher, not as part of usual care.	Pre survey 12–18 weeks pregnant. Post survey 28 and 36 weeks pregnant.	−6.00 (− 10.26, − 1.74)	Decision aid increased women’s knowledge
Montgomery et al. 2007 [20] Three-arm RCT	Pregnant women with one previous caesarean section.	Computerised analysis tool performed in birth choices clinic. IPDAS compliant	Usual routine antenatal care with birth choices clinic.	Primary: DCS, and mode of delivery. Secondary: knowledge, anxiety, and satisfaction.	United Kingdom national healthcare. Aid delivered at home or by researcher, not as part of usual care.	Pre survey between 20 and 36 weeks pregnant. Post survey after 37 weeks pregnant.	−4.20 9–6.88, − 1.54) Decision aid increased women’s knowledge	
Eden et al. 2014 [21] RCT	Pregnant women with one previous caesarean section.	Interactive computerised decision aid in English and Spanish. IPDAS compliant	ACOG paper information brochure in English and Spanish	Primary: DCS. Secondary: compared birth intentions and final delivery Outcomes.	United States of America. Private healthcare. Non and insured healthcare. Aid delivered at home or by researcher, not as part of usual care.	Pre survey anytime during pregnancy. Post survey same day.	−3.40 (− 8.49, − 1.69)	Decision aid increased women’s knowledge
Wise et al. 2019 [22] RCT	Pregnant women with one previous caesarean section attending a vaginal birth after caesarean section clinic.	Paper decision aid booklet IPDAS compliant	Usual care from specialist birth choices clinic	Primary: preferences for mode of birth and adherence to preferences. Secondary: DCS, knowledge, and maternal satisfaction.	New Zealand healthcare system. Aid read at home and followed up by specialist service.	Pre survey < 25 weeks pregnant. Post survey > 34 weeks pregnant.	0.00 (− 3.38, 3.38)	Decision aid increased women’s knowledge
Kupperman et al. 2020 [23] RCT	Pregnant women with one previous caesarean section attending a specialist birth after caesarean section clinic.	Computerised decision aid with a validated risk predictor based on demographics in English and Spanish. IPDAS compliant	Usual care from specialist birth choices clinic.	Primary: Delivery approach. Secondary: vaginal birth, maternal and neonatal morbidity as well as DCS, knowledge, decision efficacy, and decision satisfaction.	United States of America. University hospitals and community antenatal clinics. Aid delivered at home or by researcher, not as part of usual care.	Pre survey 25 weeks pregnant. Post survey 34–37 + 6 weeks pregnant.	−0.30 (− 1.67, 1.07)	Decision aid increased women’s knowledge

*RCT* Randomised control trial, *ACOG* American College of Obstetricians and Gynaecologists, *IPDAS* International patient decision aid standrads, *DCS* Decisional conflict scale

### Risk of Bias

Each trial’s methodologic quality and risk of bias was assessed by using the Jadad criteria [9] (Table 3) and scores ranged from two to three out of five. The nature of the intervention compared to usual care meant it was difficult to mask women and healthcare professionals across all ten studies. As no pre-defined scripts for control arm consultations existed, it is possible that the decision aid informed the conversations undertaken in the usual care arm which could influence the outcomes and impacts the risk of bias score. Overall, no trials were assessed as fulfilling the Jadad criteria and therefore considered high quality.

**Table 3:** Evaluation of trial quality and risk of bias

	Randomisation (2)	Blinding (2)	Account of all participants (1)	Total (n/5)
**Prunty (2008)**	2	0	1	3
**Meade (2015)**	2	0	1	3
**McGrath (2017)**	2	0	1	3
**Vigod (2019)**	2	0	1	3
**Khalifeh (2019)**	2	1	0	3
**Shorten (2005)**	2	1	0	3
**Montgomery (2007)**	1	0	1	2
**Eden (2014)**	2	1	0	3
**Wise (2019)**	2	0	0	2
**Kupperman (2020)**	2	0	0	2

### Do decision aids reduce decisional conflict in women with pre-existing morbidity?

**Fig. 2:**
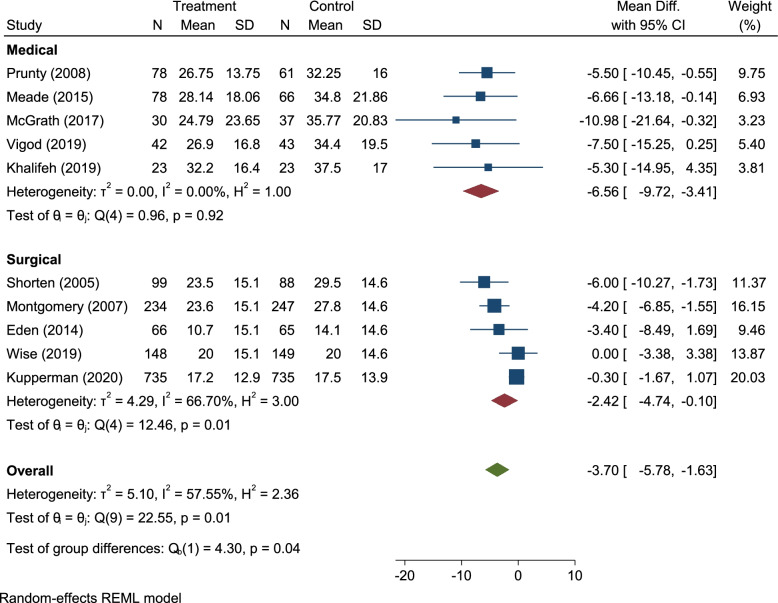
Forest plot of patient decision aids for decisional conflict. DCS, decisional conflict score; SMD, standardized mean difference. Weights are from random-effects analysis

**Fig. 3:**
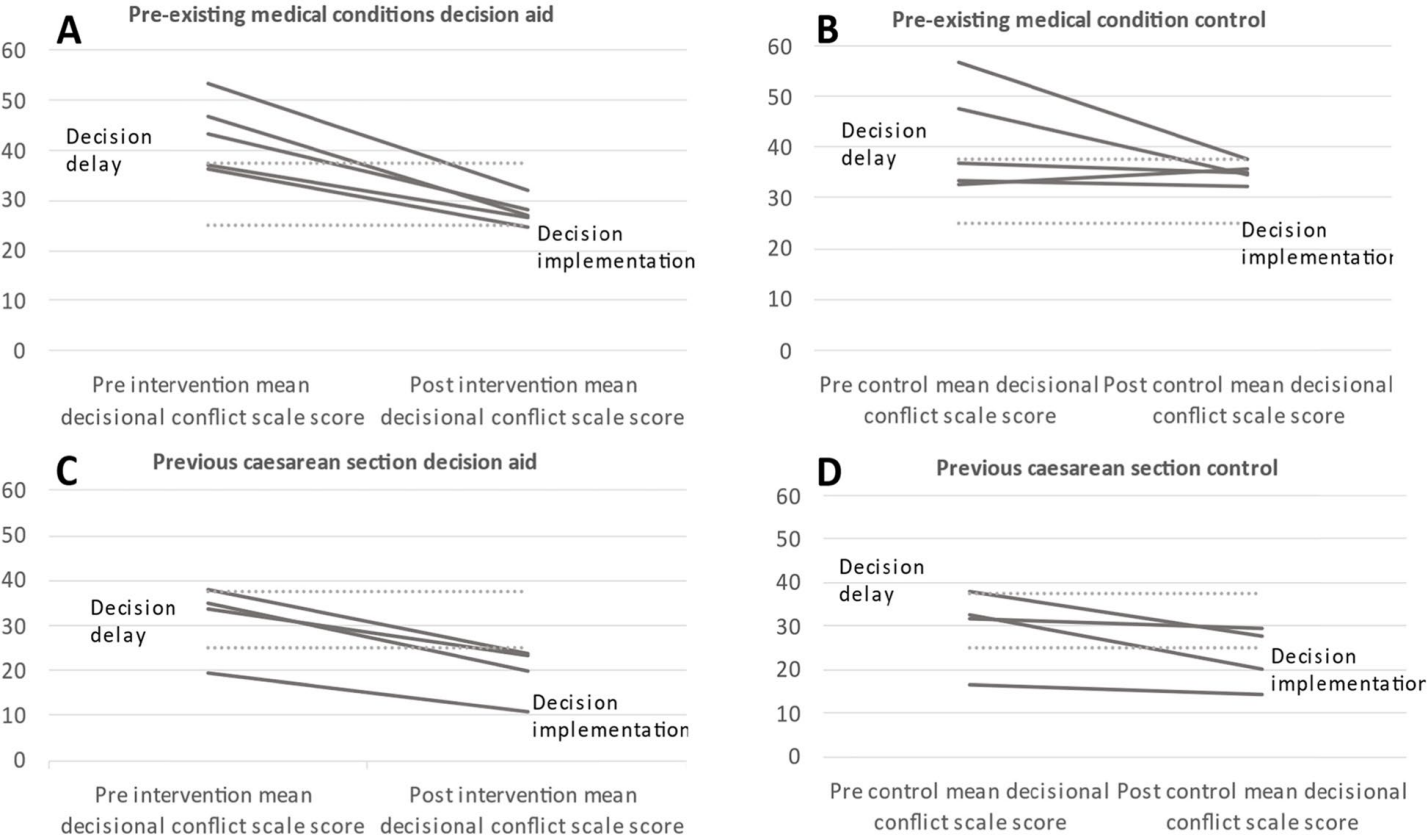
Line graph of intervention and control arm mean pre and post DCS score by sub-group. Mean DCS scores are presented alongside the score above which a person is unable to make informed decisions, and below which they can make an informed decision. **A**. Women with pre-existing medical conditions exposed to decision aid. **B**. Women with pre-existing medical condition in the control arm. **C**. Women with previous caesarean section planning mode of birth exposed to decision aid. **D**. Women with previous caesarean section planning mode of birth in the arm

All ten of the studies adopted the O’Connor DCS and were included in the meta-analysis [10]. However, Kupperman et al. did not report a pre mean DCS score in either arm of the trial and was not therefore included in the sub-analysis of pre and post mean changes. Patient decision aids additionally reduced decisional conflict by nearly 4% (Mean Difference − 3.7%, Confidence Interval 95%, − 5.9 to − 1.6) (Fig. 2) compared to the control group. Women with pre-existing medical conditions had greater reductions in decisional conflict − 6.6% (CI − 9.8 to − 3.4) compared to those who experienced previous caesarean section − 2.4% (CI − 4.8 to − 0.1). There was no heterogeneity across the studies of women with pre-existing medical conditions I-squared 0% (*p* = 0.919) (Fig. 2). Heterogeneity was detected in the studies that involved women who were making decisions about mode of birth following caesarean section I-squared 67.9% (*p* = 0.014). The overall I-squared was 59% (*p* = 0.008). Moderate heterogeneity [11] across the systematic review was influenced by two trials within the previous caesarean section sub-group, where implementation of the decision aid did not result in reductions in decisional conflict reduction [22, 23]. Initially Egger’s test found publication bias, however once pre-defined medical and surgical subgroup analysis had been performed publication bias was no longer present (supplementary file 3) [11]. Further sensitivity analysis demonstrated that women with pre-existing medical conditions were, on average, more conflicted at baseline compared to those making decisions about mode of birth following caesarean section (Fig. 3). Compared to those making decisions about mode of birth following caesarean section, women with pre-existing medical conditions appeared to be more uncertain about their pregnancy decisions at baseline and had greater reductions in decisional conflict, in keeping with clinically relevant improvements in decision-making.

### Do decision aids improve knowledge in women with pre-existing morbidity?

The ten randomised controlled trials included in the review all reported knowledge as one of their predefined outcomes. Eight out of ten studies used a validated knowledge questionnaire, [14–18, 20, 21] of which five reported significant increases in knowledge scores from the validated questionnaires in women who were exposed to the decision aid compared to the control arm [14–17, 21]. Four studies reported knowledge as a sub-score in the DCS, of which three reported significant increases in informed sub scores compared to the control arm [14, 19, 22]. In all but one trial, [18] decision aids were found to have increased women’s knowledge of their pre-existing condition compared to the control arm..

### Do decision aids improve health-related outcomes in women and infants with pre-existing morbidity?

Half of the studies included in the systematic review included health outcomes measures, including two pre-existing medical conditions studies both of which addressed anti-depressants use in pregnancy [17, 18] and three that addressed birth after a previous caesarean section [19–21]. There was no statistical difference in maternal or infant health outcomes with the implementation of decision aids in any of the five studies that reported these measures. The two pilot trials implemented decision aids that focused on antidepressants use in pregnancy measured depression and anxiety scores using a validated scale before and after the intervention; although the results were not powered to detect effect there was a trend towards those in the decision aid group having improved depressive symptoms [17, 18].

### What type of decision aids are effective for decision-making in women with pre-existing morbidity? A narrative synthesis

Both paper-based decision aids and computerised decision aids were used across the ten randomised controlled trials. Five studies trialled paper-based decision-aids between 2008 and 2019 [14–16, 19, 22] and five trialled computerised decision aids between 2014 and 2020 [17, 18, 20, 21, 23]. Two of the five computerised decision aids had aspects of personalisation, but this did not extend to personalised risk scores [20, 21]. There was minimal variation in the DCS mean difference scores of computerised and paper-based decision aids. Four studies included a decision aid in one arm of the trial and an information brouchure as part of usual care in the other arm [17, 18]. [20] [21] In this sub-group analysis reductions in mean difference DCS were greater in the decision aid arms compared to information brouchure arms [17, 18, 20]. [21] A fourth study carried out a three arm trial comprising of usual care, an online information brouchure and a personalised decision aid. Unlike the other studies, a greater reduction in decisional conflict was seen in the brouchure arm compared to personalised decision aid arm [20].

### In what circumstances are decision aids effective for decision-making in women with pre-existing morbidity? A narrative synthesis

The women recruited to the studies included in this systematic review received care in both primary and secondary care settings, from midwives, obstetricians, and physicians. Some women with perinatal mental health conditions, and some women who had had a previous caesarean section birth, received care from a specialist antenatal clinic. Two of the three studies investigating the effect of decision aids on decisional conflict in women who had had a previous caesarean section compared the decision aid to specialist service and found no difference between the two arms [22, 23]. Women with depression who received a decision aid in addition to care from a perinatal mental health specialist service reported some reductions in decisional conflict, but the effect was less marked when compared to women receiving the aid alongside routine care [17].

## Discussion

The systematic review has demonstrated that patient decision aids modestly reduce decisional conflict in women with pre-existing morbidity making pregnancy related decisions (2–7%). Women were also more knowledgeable about their condition following use of a decision aid compared to usual care that includes information brochures. Although reductions in decisional conflict were modest, on average they helped women move towards a less conflicted state, which coupled with increases in knowledge scores suggests women were more likely to make informed pregnancy related decisions. Decision aids were most beneficial when provided to women with pre-existing medical conditions who were making decisions about the safety of medication and pregnancy, as these women were generally more conflicted at baseline and had greatest reductions in mean difference. There is evidence to suggest that paper-based aids are as effective as computerised aids, but there is only limited evidence on electronic personalised decision aids. Decisions aids are likely to reduce variation amongst women making pregnancy related decisions regardless of their model of care, by standardising decision-making support across different healthcare settings.

The strength of this study includes its comprehensive search strategy and its focus on women with pre-existing morbidity who face particularly complex pregnancy decisions. Its systematic review with meta-analysis and sub-analysis of change in pre and post DCS score enables readers to understand not just the overall effectiveness of decision aids but also the clinical importance of these results. The narrative synthesis approach also enables readers to understand which type of decision aid works and in what circumstances do they work, leading to a more nuanced approach to pregnancy decision aid implementation in women with pre-existing morbidity.

The findings of this review are limited by the inherent risk of bias across all the studies. The Jadad risk of bias assessment [9] found that healthcare professionals in the control arm were not masked to the detail of the decision aid. It is therefore not possible to know whether the decision aids informed the conversations that were being undertaken in the usual care arm, reducing the comparative effectiveness of the decision aids. The lack of health outcome measures included across the studies limits the findings, but the review has identified the potential benefit of decision aids in women making decisions about medication in pregnancy which would warrant future investigation. Finally, caution must be applied to the use of mean and mean difference scores in evaluating shared decision-making interventions as the data reported in the trials do not allow us to understand individual responses and outlier perspectives.

Implementing personalised care, centred on the woman, her baby and her family, based around their needs and their decisions, where they have genuine choice, informed by unbiased information is a high priority for national and international policy makers [1]. Despite the ambition, shared decision-making implementation has been variable, particularly in the United Kingdom [5]. Women with pre-existing morbidity receive variable care as a result of disease severity, pregnancy care pathways and individual practitioner norms, values and behaviours [2]. Implementing decision aids is one strategy to improve personalised care as evidence has shown patients who have used decision aids are better informed and more active in the decision-making process [7]. This systematic review supports the implementation of decision aids into prenatal and antenatal care for women with pre-existing morbidity as they reduce unwanted variation in patient decision-making. Importantly, women with pre-existing medical conditions, on average, had greatest benefit from decision aids (by way of reductions in decisional conflict), and face some of the most complex decisions, often with uncertainties around counselling.^25^ Policy makers should work towards ensuring women with common medical conditions such as hypertension and diabetes have access to contemporary pregnancy decision aids to support personalised care and support planning.

Despite benefits associated with implementation, mean post DCS score remained high for some women in many of the studies. This is perhaps unsurprising, as all of the decision aids included in this review were delivered outside of the consultation and were not designed to facilitate personalised in-consultation care planning [24]. Low-cost paper-based tools may be as effective than electronic tools and are congruent with the idea that decision aids are a tool to be used in conjunction within consultations. Similarly, there was a lack of evidence regarding the effectiveness of the computerised patient decision analysis tool. Given the modest improvement in women’s experience of decision-making across all the trials, future studies may want to better understand whether in-consultation aids such as Option Grids, infographics, and conversation prompts are more effective at reducing decisional conflict in both specialist and non-specialist services. They may also want to better understand whether the personalising of risks and benefits by professional in-consultation is more effective and acceptable to women compared to computer-based programmes.

In addition to improving personalised care there is a national priority to improve the safety of pregnancy and birth. Recurrent reviews into maternal and perinatal mortality have identified that women with pre-existing morbidity are at greater risk of poor pregnancy outcomes [2]. There are many mechanisms that lead to poorer outcomes in this group of women, only some of which are preventable [2]. Medication adherence is a modifiable mechanism that can improve outcomes in women with pre-existing medical conditions [25]. Outside of pregnancy there is some evidence that has shown patient decision aids improve decision-making and health outcomes in patients with chronic conditions such as hypertension [26]. This systematic review has identified pilot data that suggests implementing decision aids in women with pre-existing medical conditions may improve health outcomes mediated by reductions in unwanted variance in medication behaviours, which warrants further investigation. Future research may wish to understand whether implementation of decision aids for pharmacological decisions in pregnancy improve pregnancy outcomes and the mechanisms of action that are involved.

In conclusion, patient decision aids support personalised care planning and informed decision-making in women with pre-existing morbidity, although the effect may be modest. Women with pre-existing medical morbidity are likely to benefit from decision aids because of high levels of existing conflict and the effectiveness of decision aids in this group. Further, adoption of aids in this population may lead to improve adherence and health outcomes. It is likely that the adoption of a co-design approach to the development of decision aids would result in more effective tools, as women themselves are best placed to identify their decisional needs. Future research should also consider whether in-consultation aids better support personalised care and informed decision-making.

## Supplementary Information

**Additional file 1.**

**Additional file 2.**

**Additional file 3.**

**Additional file 4.**

**Additional file 5.**

## Data Availability

The datasets generated and/or analysed during the current study are not publicly available due to the secondary nature of this research but are available from the corresponding author on reasonable request.
