# Supplementary material for: The effectiveness of decision aids for pregnancy related decision-making in women with pre-pregnancy morbidity; systematic review and meta-analysis
Source: BMC Pregnancy Childbirth. 2022 Jan 29;22:81. doi: 10.1186/s12884-022-04402-x (PMC8801107; doi:10.1186/s12884-022-04402-x)
Supplement: Supplementary file 1 — Additional file 1. [file 12884_2022_4402_MOESM1_ESM.pdf]

Systematic review of the effectiveness of decision aids for pregnancy related decision-making in women with pre-pregnancy morbidity

REGISTRATION

Registered on PROSPERO, registration number:

AUTHORS

Rebecca Whybrow, Louise M Webster, Jane Sandall, Lucy C Chappell

Department of Women and Children's Health,

School of Life Course Sciences,

King's College London10th floor North Wing

St Thomas' Hospital

SE1 7EH

Email: Rebecca.whybrow@kcl.ac.uk

Contributions:

RW wrote the protocol and LMW, JS and LCC edited it.

AMENDMENTS

This is the first version of this protocol and any amendments will be added to the PROSPERO website.

SUPPORT

This review was supported by Kings College London

INTRODUCTION

Women entering pregnancy with pre-existing morbidity arising from medical conditions or previous surgery are at higher risk of poor pregnancy outcomes compared to women without such morbidity. These women often face complex pregnancy-related decision-making that may be characterized by conflicting maternal and perinatal priorities. Decision aids provide individuals with information about the choices that need to be made as well as a process to help them make informed decisions regarding the balance of benefit and risk. It is uncertain whether decision aids are effective in reducing decisional conflict and in improving maternal and neonatal outcomes in women with pre-pregnancy morbidity.

OBJECTIVES

To perform a systematic review of randomised controlled trials (RCTs) to evaluate the following questions:

- Do decision aids reduce decisional conflict in women with pre-existing morbidity?
- Do decision aids improve knowledge in women with pre-existing morbidity?
- Do decision aids improve health-related outcomes in women with pre-existing morbidity?
- Do decision aids improve health-related outcomes in infants of mothers with pre-existing morbidity?
- What type of decision-aids are effective for decision-making in women with pre-existing morbidity?
- In what circumstances are decision-aids effective for decision-making in women with pre-existing morbidity?

METHODS

Population

The population is women with a pre-pregnancy morbidity (medical or surgical); this encompasses medical conditions where the condition may affect the course of the pregnancy or where the pregnancy affects the course of the disease and those with prior surgery that may affect the course of the pregnancy and birth or where surgical morbidity may be affected by pregnancy and birth.

Types of Intervention

- Any decision aid that has been compared against no intervention or usual care
- Any decision aid that has been compared against another decision aid with pre and post decision aid measures

Eligibility criteria *(Published and unpublished RCTs in any language will be assessed for eligibility)*

- Women with a pre-existing medical morbidity
- Women with pre-existing psychological morbidity
- Women with pre-existing surgical morbidity

Exclusion criteria

- Any study designs other than RCT
- Any decision aids that do not meet the International Patient Decision Aid Standards (IPDAS)

Outcomes of interest

Main outcomes:

- Process
    - Measures of decisional conflict
    - Measures of maternal knowledge

Secondary outcomes:

- Maternal
    - Adherence to chosen option
    - Adverse events and drug side effects
    - Anxiety
    - Attainment of intended choice
    - Choice behavior
    - Depression
    - Maternal death
    - Health related outcome specific to condition
        - Mode of delivery
        - Estimated blood loss at delivery
        - Intensive Therapy Unit/High Dependency Unit admission nights
        - Depression
        - Anxiety
        - Hypertension or pre-eclampsia
        - Other severe maternal morbidity: Disseminated Intravascular Coagulation, Diabetic Ketoacidosis, Hypoglycemia, Acute Kidney Injury, Acute Liver Injury, Stroke, Seizures, Placental abruption

- Length of stay antenatal, length of stay postnatal and readmission mother

- Fetal/Neonatal:
  - Intrauterine fetal loss: (categorized as <24 weeks' gestation or >= 24 weeks' gestation)
  - Neonatal Death (death within the first 28 days of life)
  - Preterm birth (<37 weeks and subdivided into <34 weeks wherever possible)
  - Small for gestational age (SGA) babies and large for gestational age (subdivided into birth centiles <10th centile, <3rd centile where possible and >90th centile)
  - Apgar score at 5 minutes
  - Length of postnatal stay
  - Neonatal unit admission
  - Any neonatal morbidity thought to be related to maternal medication such as hypo/hypertension, hypoglycemia, etc.
  - Fetal abnormality

The primary outcomes chosen are process outcomes that establish the effectiveness of the interventions to achieve satisfactory decision-making. The secondary outcomes establish whether the decisions made had an effect on maternal and fetal health outcomes.

Search strategy

The following databases will be searched:

- Medline (via OVID)
- Embase (via OVID)
- Cochrane Trials Register
- CINAHL

Databases will be searched from their earliest entries until 01/10/2018 and will be updated immediately prior to sending for publication. No language or country restriction will be used in searches. Searches will be adapted to each database and details of each planned strategy are listed in the appendix.

In addition any currently registered relevant clinical trials will be searched for via:

- Clinicaltrials.gov
- ISRCTN.com

Other grey literature will be sought by reviewing thesis titles from WorldCat Dissertations and Theses database. The Prospero and Cochrane databases will be reviewed for registered protocols.

Study records and data extraction

The titles and abstracts generated from the database searches will be independently screened by two authors. If either author considers the study to meet inclusion criteria it will be included for second round screening where full text assessment will take place. Any disagreements will be initially resolved between the two reviewers but where agreement cannot be reached it will be resolved by involving a third independent reviewer. Any foreign language abstracts will be translated. Data from eligible papers will be manually extracted and entered into a standard extraction table independently by the two primary reviewers. Data from all studies will then be collectively tabulated. Where there is lack of clarity in data or study design, every effort will be made to contact the authors of the trial for further information.

Study quality assessment

Each individual trial will be quality assessed by one author using the Cochrane Collaboration Risk of Bias tool and then checked by a second author. Where there are any disagreements, they will be resolved between themselves and when agreement cannot be made by discussion with a third author. The following areas of each study will be considered for bias:

- Sequence generation
- Allocation concealment
- Blinding of participants, personnel and outcome assessors
- Incomplete outcome data
- Selective outcome reporting
- Any other sources of bias (including funding source)

Sensitivity analysis will be performed.

Data will be collected and tabulated on:

- Definition of decision aid
- Pre-pregnancy morbidity (medical, psychological and surgical)
- Context (country, health system, finance).
- Participant characteristics (morbidity, primips, multips, both) gestation of pregnancy, morbidity, socio-demographic information).
- Intervention characteristics including: Setting (at home or in consultation), mode of delivery (paper or electronic), type of provider (health professional or a lay person).

- Content (of aid).
- Implementation (frequency/duration, intervention fidelity, adaptation)
- Characteristics of usual care in comparison group
- Funding source

Data synthesis

The review will be performed in line with PRISMA guidelines. For all outcomes the analysis will be conducted on an intention to treat basis. If a study contains only per-protocol data for a particular endpoint, it will be excluded from the main analysis. Meta-analysis will be undertaken where there is more than one study with data suitable for inclusion. When there is only one study, the estimates from that study will be presented. For continuous data, we will use the mean difference with 95% CI if outcomes are measured in the same way between trials. We will use the standardised mean difference with 95% CI to combine outcomes from trials that measure the same outcome but use different scales. In the event of important significant treatment effects, the Number Needed to Treat (NNT) or Number Needed to Harm (NNH) will also be given. Conventional significance will be at the usual 5% level (2-sided).

Dealing with missing data

For included studies, we will note levels of attrition. We will explore the impact of including studies with high levels of missing data in the overall assessment of treatment effect by using sensitivity analysis. For all outcomes, we will carry out analyses, as far as possible, on an intention-to-treat basis, i.e. we will attempt to include all participants randomised to each group in the analyses, and all participants will be analysed in the group to which they were allocated, regardless of whether or not they received the allocated intervention. The denominator for each outcome in each trial will be the number randomised minus any participants whose outcomes are known to be missing. We will include outcomes from trials where attrition is over 20% but conduct a sensitivity analysis to identify the effects of attrition bias on effect estimates.

Dealing with heterogeneity

Heterogeneity of results between studies will be tested using a Chi-squared test. Significant heterogeneity will be assessed using $Tau^{2,}$ I^2 and by visual inspection of the forest plot. Where there are sufficient studies of different populations, entry criteria or treatments, meta-regression and subgroup analysis will be used to investigate the differences between study results. Meta-regression will be performed with reported variables that are suspected to influence efficacy of treatment.

Publication bias will be investigated using Egger's test and funnel plots. If there are 10 or more studies in the meta-analysis, we will investigate reporting biases (such as publication bias) using funnel plots. We will assess funnel plot asymmetry visually. If asymmetry is suggested by a visual assessment, we will perform exploratory analyses to investigate it.

Subgroup analysis

- Medical condition
- Psychological condition
- Surgical condition
- Type of decision aid
- Parity

Where data collected are insufficient for quantitative synthesis, they will be tabulated for presentation and explored in the discussion. If outcomes of interest are not able to be quantitatively synthesised, recommendations for the definitive trial to obtain these data will be made in the study conclusions.

Confidence in cumulative evidence

For each outcome of interest presented in a summary of findings table, the quality of the body of evidence will be assessed using the Grading of Recommendations Assessment, Development and Evaluation (GRADE) system, in line with recommendations from the GRADE working group. Quality of evidence will be determined as high, moderate, low or very low through assessing:

- risk of bias
- directness
- consistency of results
- precision
- publication bias
- magnitude of effect
- dose-response relationship

Confidence of the effect size of intervention on outcomes of interest will be based on this assessment.

APPENDIX

a. Search strategy for Medline (via OVID platform)

1. Pregnancy/
2. exp Prenatal Diagnosis/
3. exp Parturition/
4. exp Decision Support Techniques/
5. exp Decision Making
6. exp Randomized Controlled Trials as Topic/
7. exp Clinical Trial/
8. 1 or 2 or 3
9. 4 or 5
10. 6 or 7
11. 8 and 9 and 10

b. Search strategy for Embase (via OVID platform)

1. exp pregnancy/
2. exp prenatal diagnosis/
3. exp birth/
4. exp decision support system/
5. exp decision making
6. randomized controlled trial/
7. clinical trial/
8. 1 or 2 or 3
9. 4 or 5
10. 6 or 7
11. 8 and 9 and 10

c. Search strategy for CINHAL

1. TX Pregnancy
2. TX Prenatal Diagnosis
3. TX Parturition
4. TX Decision Support Techniques
5. TX Decision Making
6. TX Randomized Controlled Trials
7. TX Clinical Trial/
8. 1 or 2 or 3

9. 4 or 5

10. 6 or 7

11. 8 and 9 and 10

d. Search strategy for Cochrane Library

Pregnancy and Decision Support
