# Supplementary material for: The effectiveness of decision aids for pregnancy related decision-making in women with pre-pregnancy morbidity; systematic review and meta-analysis
Source: BMC Pregnancy Childbirth. 2022 Jan 29;22:81. doi: 10.1186/s12884-022-04402-x (PMC8801107; doi:10.1186/s12884-022-04402-x)
Supplement: Supplementary file 2 — Additional file 2. [file 12884_2022_4402_MOESM2_ESM.pdf]

# User Manual – Decisional Conflict Scale

## *Table of Contents*


1. Definition ....................................................................................................................1
2. Conceptual Framework .....................................................................................................2
3. Comparison of Versions of the Decisional Conflict Scale (DCS) [Table] ...........................................3
4. Versions of the Decisional Conflict Scale (DCS)
    4.1 Traditional DCS: Statement format 16 item 5 response categories .........................................4
        Sample Tool
        Scoring and Interpretation
        Psychometric Properties
        Applications Using this Tool
        Other Languages
        Availability
        Suggested Citation
    4.2 Question Format DCS: 16 item 5 response categories ...........................................................7
        Sample Tool
        Scoring and Interpretation
        Psychometric Properties
        Applications Using this Tool
        Other Languages
        Availability
        Suggested Citation
    4.3 Question Format DCS: 10 item 3 response categories ...........................................................9
        Sample Tool
        Scoring and Interpretation
        Psychometric Properties
        Applications Using this Tool
        Other Languages
        Availability
        Suggested Citation
    4.4 SURE tool version for clinical practice: 4 items 2 response categories .................................12
        Sample Tool
        Scoring and Interpretation
        Psychometric Properties
        Applications Using this Tool
        Other Languages
        Availability
        Suggested Citation
5. Annotated References ......................................................................................................15


## *1. Definition*

The decisional conflict scale measures personal perceptions of : a) uncertainty in choosing options; b) modifiable factors contributing to uncertainty such as feeling uninformed, unclear about personal values and unsupported in decision making; and c) effective decision making such as feeling the choice is informed, values-based, likely to be implemented and expressing satisfaction with the choice.

## 2. *Conceptual Framework*

The conceptual framework guiding the scale development is reported in detail elsewhere [1]. It was derived from the construct of decisional conflict developed by Janis and Mann [16] and refined as a diagnosis by the North American Nursing Diagnosis Association [17]. Decisional conflict is a state of uncertainty about a course of action. Such uncertainty is more likely when a person is confronted with decisions involving risk or uncertainty of outcomes, when high-stakes choices with significant potential gains and losses are entertained, when there is a need to make value tradeoffs in selecting a course of action, or when anticipated regret over the positive aspects of rejected options is probable. The main behavioural manifestations of decisional conflict include verbalized uncertainty about choices, verbalization of the undesired consequences of alternatives; vacillation between choices, and delayed decision making. Minor manifestations include verbalized distress while attempting decision making, self-focusing, physical signs of distress or tension, and questioning personal values and beliefs while attempting to make a decision.

Although decisional conflict occurs as a consequence of the difficulty inherent in the type of decision being made, several modifiable cognitive, affective and social factors can exacerbate the perceived uncertainty. Uncertainty is greater when a person: 1) feels uninformed about the alternatives, benefits and risks; 2) is unclear about personal values; 3) feels unsupported in making a choice or pressured to choose on course of action.

Decisional conflict can be lowered with decision supporting interventions. Information about options, benefits, risks, and side effects can make people feel more informed. Values can be clarified using strategies such as: describing outcomes in sufficient detail (including physical, emotional, and social impacts) to better judge their value; and asking patients to rate the personal importance of outcomes. People may feel more supported in decision making if they are guided or coached in the steps of deliberation and shared decision making. As a consequence, their uncertainty stemming from these modifiable factors may decline, and they may feel they have made a better decision. By better decision, they may feel that they have made a more informed value-based decision, are more likely to stick with their choice, and are more satisfied with the decision.

There is considerable empirical data to support the effects of decision supporting interventions on decisional conflict and its related modifiable constructs [1,3].

## *3. Comparison of Versions of the Decisional Conflict Scale (DCS)*

There are 4 versions of the scale: 1 for clinical practice (see page 12) and 3 for research. This table compares the research versions. More details on these three versions are also provided on subsequent pages.

| **A. STATEMENT FORMAT**<br>**16 items**<br>**5 response categories**<br>**statements**<br>• Used in most studies to date<br>• Many respondents like this version because it is personal<br>• Those with limited response skills find it harder to respond to | **B. QUESTION FORMAT**<br>**16 items**<br>**5 response categories**<br>**questions**<br>• Easier response format<br>• More precision than 3 item response format<br>• Least tested: now being tested with hundreds of patients | **C. LOW LITERACY**<br>**10 items**<br>**3 response categories**<br>**questions**<br>• Response format most useful for people with limited reading or response skills<br>• Willing to forgo precision<br>• Second most tested version |
|---|---|---|
| **RESPONSE FORMATS** | | |
| **strongly agree, agree, neither agree or disagree, disagree, strongly disagree** | **yes, probably yes, unsure, probably no, no** | **yes, no, unsure** |
| **INFORMED SUBSCALE** | | |
| I know which options are available to me. | Do you know which options are available to you? | Do you know which options are available to you? |
| I know the benefits of each option. | Do you know the benefits of each option? | Do you know the benefits of each option? |
| I know the risks and side effects of each option. | Do you know the risks and side effects of each option? | Do you know the risks and side effects of each option? |
| **VALUES CLARITY SUBSCALE** | | |
| I am clear about which benefits matter most to me. | Are you clear about which benefits matter most to you? | Are you clear about which benefits matter most to you? |
| I am clear about which risks and side effects matter most. | Are you clear about which risks and side effects matter most to you? | Are you clear about which risks and side effects matter most to you? |
| I am clear about which is more important to me (the benefits or the risks and side effects). | Are you clear about which is more important to you (the benefits or the risks and side effects)? | |
| **SUPPORT SUBSCALE** | | |
| I have enough support from others to make a choice. | Do you have enough support from others to make a choice? | Do you have enough support from others to make a choice? |
| I am choosing without pressure from others. | Are you choosing without pressure from others? | Are you choosing without pressure from others? |
| I have enough advice to make a choice. | Do you have enough advice to make a choice? | Do you have enough advice to make a choice? |
| **UNCERTAINTY SUBSCALE** | | |
| I am clear about the best choice from me. | Are you clear about the best choice for you? | Are you clear about the best choice for you? |
| I feel sure about what to choose. | Do you feel sure about what to choose? | Do you feel sure about what to choose? |
| This decision is easy for me to make. | Is this decision easy for you to make? | |
| **EFFECTIVE DECISION SUBSCALE** | | |
| I feel I have made an informed choice. | Do you feel you have made an informed choice? | |
| My decision shows what is important to me. | Does your decision show what is important to you? | |
| I expect to stick with my decision. | Do you expect to stick with your decision? | |
| I am satisfied with my decision. | Are you satisfied with your decision? | |

## *4. Versions of the Decisional Conflict Scale (DCS)*

### 4.1 Traditional Decisional Conflict Scale (DCS) - Statement Format: 16 item 5 response categories

This is our most tested version. Many people like the personal response format. However it is more difficult to respond to than questions in those with limited reading and response skills.
Note: We always precede the DCS with an option preference question, which is not included in scoring. [See item 'A' below].

## 4.1.1 Scale
## My difficulty in making this choice

A. Which [insert treatment/screening] option do you prefer? Please check ☑ one.

- ☐ **[Option 1]**
- ☐ **[Option 2]**
- ☐ **[Option 3]**
- ☐ Unsure

B. Considering the option you prefer, please answer the following questions:

| | **Strongly Agree** [0] | **Agree** [1] | **Neither Agree Or Disagree** [2] | **Disagree** [3] | **Strongly Disagree** [4] |
|---|---|---|---|---|---|
| 1. I know which options are available to me. | ☐ | ☐ | ☐ | ☐ | ☐ |
| 2. I know the benefits of each option. | ☐ | ☐ | ☐ | ☐ | ☐ |
| 3. I know the risks and side effects of each option. | ☐ | ☐ | ☐ | ☐ | ☐ |
| 4. I am clear about which benefits matter most to me. | ☐ | ☐ | ☐ | ☐ | ☐ |
| 5. I am clear about which risks and side effects matter most. | ☐ | ☐ | ☐ | ☐ | ☐ |
| 6. I am clear about which is more important to me (the benefits or the risks and side effects). | ☐ | ☐ | ☐ | ☐ | ☐ |
| 7. I have enough support from others to make a choice. | ☐ | ☐ | ☐ | ☐ | ☐ |
| 8. I am choosing without pressure from others. | ☐ | ☐ | ☐ | ☐ | ☐ |
| 9. I have enough advice to make a choice. | ☐ | ☐ | ☐ | ☐ | ☐ |
| 10. I am clear about the best choice for me. | ☐ | ☐ | ☐ | ☐ | ☐ |
| 11. I feel sure about what to choose. | ☐ | ☐ | ☐ | ☐ | ☐ |
| 12. This decision is easy for me to make. | ☐ | ☐ | ☐ | ☐ | ☐ |
| 13. I feel I have made an informed choice. | ☐ | ☐ | ☐ | ☐ | ☐ |
| 14. My decision shows what is important to me. | ☐ | ☐ | ☐ | ☐ | ☐ |
| 15. I expect to stick with my decision. | ☐ | ☐ | ☐ | ☐ | ☐ |
| 16. I am satisfied with my decision. | ☐ | ☐ | ☐ | ☐ | ☐ |

**Decisional Conflict Scale** © AM O'Connor, 1993, revised 2005

### 4.1.2 Scoring and interpretation

Items are given a score value of:

0 = ‘strongly agree’; 1 = ‘agree’; 2 = ‘neither agree nor disagree’; 3 = ‘disagree’; 4 = ‘strongly disagree’.

TOTAL SCORE

16 items [items **1-16** inclusive] are: a) summed; b) divided by 16; and c) multiplied by 25.
Scores range from 0 [no decisional conflict] to 100 [extremely high decisional conflict].

UNCERTAINTY SUBSCORE

3 items [ **10, 11, 12** ] are: a) summed; b) divided by 3; and c) multiplied by 25.
Scores range from 0 [feels extremely certain about best choice] to 100 [feels extremely uncertain about best choice].

INFORMED SUBSCORE

3 items [ **1, 2, 3** ] are: a) summed; b) divided by 3; and c) multiplied by 25.
Scores range from 0 [feels extremely informed] to 100 [feels extremely uninformed].

VALUES CLARITY SUBSCORE

3 items **[ 4, 5, 6** ] are: a) summed; b) divided by 3; and c) multiplied by 25.
Scores range from 0 [feels extremely clear about personal values for benefits & risks/side effects] to 100 [feels extremely unclear about personal values]

SUPPORT SUBSCORE

3 items [ **7, 8, 9** ] are: a) summed; b) divided by 3; and c) multiplied by 25.
Scores range from 0 [feels extremely supported in decision making] to 100 [feels extremely unsupported in decision making].

EFFECTIVE DECISION SUBSCORE

4 items [ **13, 14, 15, 16** ] are: a) summed; b) divided by 4; and c) multiplied by 25.
Scores range from 0 [good decision] to 100 [bad decision].

**NOTE: The Cochrane systematic review of trials of patient decision aids [3] uses this scoring method; other papers may present scores ranging from 1 [low decisional conflict] to 5 [high decisional conflict]. If the SCORE or SUBSCORE is reported as a percentage or on a 0-100 scale no conversion is required.**

**If the average SCORE or SUBSCORE is reported on the 1-5 scale then to convert these scores to the equivalent 0-100 scale: a) subtract 1 from the score; b) then multiply by 25.**

**If the SCORE or SUBSCORE is reported as a sum of items that used the 1-5 scale then to convert these scores to the equivalent 0-100 scale: a) divide the score by the number of items summed; b) then subtract 1; c) then multiple by 25.**

### 4.1.3 Psychometric Properties

**Reliability:**

- test-retest correlations and Cronback alpha coefficients exceed 0.78.

**Construct Validity:**

- correlated to related constructs of knowledge, regret, and discontinuance.
- discriminates between known groups: those who make and delay decisions (effect size [ES] ranges 0.4 to 0.8).

**Responsive to change:**

- in before/after studies of decision supporting interventions, effect size ranges from 0.4 to 1.2 for the total scale.
- discriminates between different decision supporting interventions:
    - Informed subscale consistently discriminates (ES 0.3 to 0.4).
    - Total score and other sub-scores less consistent [especially when decision supporting intervention compared to alternative intervention rather than to usual care controls
        - total scale ES: 0.2 to 0.3
        - uncertainty scale: 0.06 to 0.3
        - unclear values ES: 0.3 to 0.4
        - unsupported ES: 0.0 to 0.3
        - quality of choice: 0.2 to 0.3

**Predictive validity:**
Sun [18] found that for every unit increase in the DCS, people were:

- 59 times more likely to change their mind
- 23 times more likely to delay their decision
- 5 times more likely to express decisional regret
- 3 times more likely to fail a knowledge test on options

Gattelari and Ward [19] found that for every unit increase in DCS, patients were 19% more likely to blame their doctor for bad outcomes. Decisional conflict was an independent predictor of blame, separate from other predictors such as knowledge scores and age of the patient.

**Meaningful differences:**

- we usually base sample sizes on detecting an effect size of 0.30 to 0.40;
- scores lower than 25 are associated with implementing decisions; scores exceeding 37.5 are associated with decision delay or feeling unsure about implementation.

### 4.1.4 Applications using this tool

The scale has been used in more than 30 studies for numerous decisions.

### 4.1.5 Availability

**You may use any of these scales at no cost without permission.**

These tools are protected by copyright but are freely available for you to use, provided you cite the reference in any questionnaires or publications.

### 4.1.6 Suggested Citations

O'Connor AM. Validation of a decisional conflict scale. Med Dec Making 1995; 15(1): 25-30. The classic psychometric paper.

O'Connor AM. User Manual - Decisional Conflict Scale (16 item statement format) [document on the Internet]. Ottawa: Ottawa Hospital Research Institute; © 1993 [updated 2010; cited YYYY MM DD]. 16 p. Available from http://decisionaid.ohri.ca/docs/develop/User_Manuals/UM_Decisional_Conflict.pdf.

### *4.2*. Question Format Decisional Conflict Scale (DCS) - 16 item 5 response categories

This version is being tested by the Foundation for Informed Medical Decision Making as part of a standardized suite of decision process quality and decision quality measures using IPDAS Collaboration.
Note: We always precede the DCS with an option preference question, which is not included in scoring. [See item 'A' below].

### 4.2.1 Scale

## My difficulty in making this choice

A. Which [insert treatment/screening] option do you prefer? Please check ☑ one.

a. ☐ **[Option 1]**
b. ☐ **[Option 2]**
c. ☐ **[Option 3]**
d. ☐ Unsure

B. Considering the option you prefer, please answer the following questions:

| | **Yes** [0] | **Probably yes** [1] | **Unsure** [2] | **Probably no** [3] | **No** [4] |
|---|---|---|---|---|---|
| 1. Do you know which options are available to you? | ☐ | ☐ | ☐ | ☐ | ☐ |
| 2. Do you know the benefits of each option? | ☐ | ☐ | ☐ | ☐ | ☐ |
| 3. Do you know the risks and side effects of each option? | ☐ | ☐ | ☐ | ☐ | ☐ |
| 4. Are you clear about which benefits matter most to you? | ☐ | ☐ | ☐ | ☐ | ☐ |
| 5. Are you clear about which risks and side effects matter most to you? | ☐ | ☐ | ☐ | ☐ | ☐ |
| 6. Are you clear about which is more important to you (the benefits or the risks and side effects)? | ☐ | ☐ | ☐ | ☐ | ☐ |
| 7. Do you have enough support from others to make a choice? | ☐ | ☐ | ☐ | ☐ | ☐ |
| 8. Are you choosing without pressure from others? | ☐ | ☐ | ☐ | ☐ | ☐ |
| 9. Do you have enough advice to make a choice? | ☐ | ☐ | ☐ | ☐ | ☐ |
| 10. Are you clear about the best choice for you? | ☐ | ☐ | ☐ | ☐ | ☐ |
| 11. Do you feel sure about what to choose? | ☐ | ☐ | ☐ | ☐ | ☐ |
| 12. Is this decision easy for you to make? | ☐ | ☐ | ☐ | ☐ | ☐ |
| 13. Do you feel you have made an informed choice? | ☐ | ☐ | ☐ | ☐ | ☐ |
| 14. Does your decision show what is important to you? | ☐ | ☐ | ☐ | ☐ | ☐ |
| 15. Do you expect to stick with your decision? | ☐ | ☐ | ☐ | ☐ | ☐ |
| 16. Are you satisfied with your decision? | ☐ | ☐ | ☐ | ☐ | ☐ |

**Decisional Conflict Scale** © AM O'Connor, 1993, revised 2005

### 4.2.2 Scoring and Interpretation

Items are given a score value of:
0 = 'yes'; 1 = 'probably yes'; 2 = 'unsure'; 3 = 'probably no'; 4 = 'no'.

TOTAL SCORE
16 items [items **1-16** inclusive] are: a) summed; b) divided by 16; and c) multiplied by 25.
Scores range from 0 [no decisional conflict] to 100 [extremely high decisional conflict].

UNCERTAINTY SUBSCORE
3 items [ **10, 11, 12** ] are: a) summed; b) divided by 3; and c) multiplied by 25.
Scores range from 0 [feels extremely certain about best choice] to 100 [feels extremely uncertain about best choice].

INFORMED SUBSCORE
3 items [ **1, 2, 3** ] are: a) summed; b) divided by 3; and c) multiplied by 25.
Scores range from 0 [feels extremely informed] to 100 [feels extremely uninformed].

VALUES CLARITY SUBSCORE
3 items [ **4, 5, 6** ] are: a) summed; b) divided by 3; and c) multiplied by 25.
Scores range from 0 [feels extremely clear about personal values for benefits & risks/side effects] to 100 [feels extremely unclear about personal values]

SUPPORT SUBSCORE
3 items [ **7, 8, 9** ] are: a) summed; b) divided by 3; and c) multiplied by 25.
Scores range from 0 [feels extremely supported in decision making] to 100 [feels extremely unsupported in decision making].

EFFECTIVE DECISION SUBSCORE
4 items [ **13, 14, 15, 16** ] are: a) summed; b) divided by 4; and c) multiplied by 25.
Scores range from 0 [good decision] to 100 [bad decision].

### 4.2.3 Psychometric Properties

We will have psychometric data on the scale's performance shortly.

### 4.2.4 Applications using this Tool

The scale is currently being tested in several clinical services with large scale populations in New Hampshire [breast cancer, back surgery, hip and knee surgery, PSA testing].

### 4.2.5 Availability

**You may use any of these scales at no cost without permission.**

These tools are protected by copyright but are freely available for you to use, provided you cite the reference in any questionnaires or publications.

### 4.2.6 Suggested Citation

O'Connor AM. User Manual - Decisional Conflict Scale (16 item question format) [document on the Internet]. Ottawa: Ottawa Hospital Research Institute; © 1993 [updated 2010; cited YYYY MM DD]. 16 p. Available from http://decisionaid.ohri.ca/docs/develop/User_Manuals/UM_Decisional_Conflict.pdf .

### 4.3 **Question Format DCS - 10 item 3 response categories**

This version is recommended for those with limited reading or response skills.
Note: We always precede the DCS with an option preference question, which is not included in scoring. [See item 'A' below].

### 4.3.1 Scale

## My difficulty in making this choice

A. Which [insert treatment/screening] option do you prefer? Please check ☑ one.

a. ☐ **[Option 1]**
b. ☐ **[Option 2]**
c. ☐ **[Option 3]**
d. ☐ Unsure

B. Considering the option you prefer, please answer the following questions:

| | **Yes** [0] | **Unsure** [2] | **No** [4] |
|---|---|---|---|
| 1. Do you know which options are available to you? | ☐ | ☐ | ☐ |
| 2. Do you know the benefits of each option? | ☐ | ☐ | ☐ |
| 3. Do you know the risks and side effects of each option? | ☐ | ☐ | ☐ |
| 4. Are you clear about which benefits matter most to you? | ☐ | ☐ | ☐ |
| 5. Are you clear about which risks and side effects matter most to you? | ☐ | ☐ | ☐ |
| 6. Do you have enough support from others to make a choice? | ☐ | ☐ | ☐ |
| 7. Are you choosing without pressure from others? | ☐ | ☐ | ☐ |
| 8. Do you have enough advice to make a choice? | ☐ | ☐ | ☐ |
| 9. Are you clear about the best choice for you? | ☐ | ☐ | ☐ |
| 10. Do you feel sure about what to choose? | ☐ | ☐ | ☐ |

**Decisional Conflict Scale** © AM O'Connor, 1993, revised 2005

### 4.3.2 Scoring and Interpretation

Items are given a score value of:
0 = 'yes'; 2 = 'unsure'; 4 = 'no'.

TOTAL SCORE
10 items [items **1-10** inclusive] are: a) summed; b) divided by 10; and c) multiplied by 25.
Scores range from 0 [no decisional conflict] to 100 [extremely high decisional conflict].

UNCERTAINTY SUBSCORE
2 items [ **9, 10** ] are: a) summed; b) divided by 2; and c) multiplied by 25.
Scores range from 0 [feels extremely certain about best choice] to 100 [feels extremely uncertain about best choice].

INFORMED SUBSCORE

3 items [ **1, 2, 3** ] are: a) summed; b) divided by 3; and c) multiplied by 25.
Scores range from 0 [feels extremely informed] to 100 [feels extremely uninformed].

VALUES CLARITY SUBSCORE

2 items [ **4, 5** ] are: a) summed; b) divided by 2; and c) multiplied by 25.
Scores range from 0 [feels extremely clear about personal values for benefits & risks/side effects] to 100 [feels extremely unclear about personal values]

SUPPORT SUBSCORE

3 items [ **6, 7, 8** ] are: a) summed; b) divided by 3; and c) multiplied by 25.
Scores range from 0 [feels extremely supported in decision making] to 100 [feels extremely unsupported in decision making].

### 4.3.3 Psychometric Properties

**English**: The scale has been tested with 63 women considering breast cancer options. Alpha coefficient was 0.86. The scale is responsive to change from baseline (time 1) to the time following use of a video decision aid (T2) and counseling about options (T3).

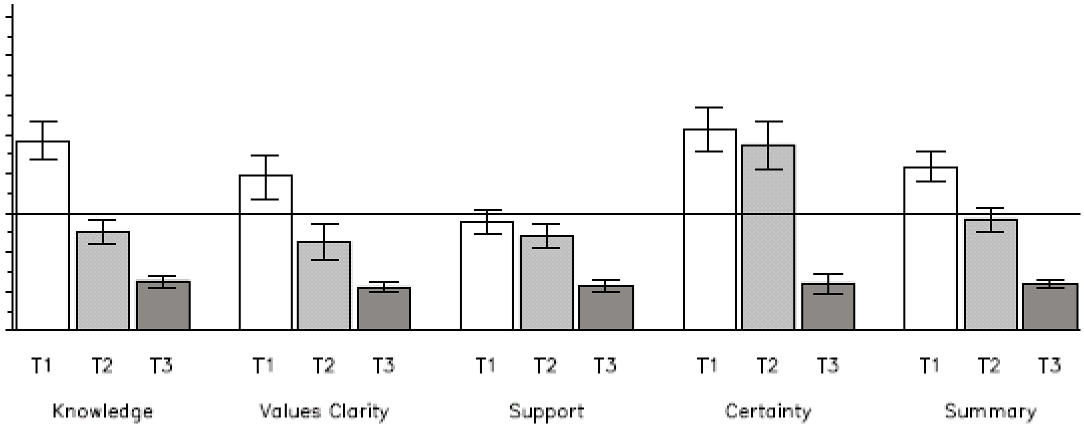

**Spanish:** The *low literacy version* has been tested with Spanish speaking women in disadvantaged areas in Chile. The alpha coefficient was 0.72 and the scale discriminated significantly between those who were: a) actively deliberating among options; and b) not contemplating options or had already made their choice.

### 4.3.4 Applications using this Tool

The scale has been used in low literacy populations in Canada (Ottawa), USA (New Hampshire, Washington DC, Texas) and Chile.

### 4.3.5 Availability

**You may use any of these scales at no cost without permission.**

These tools are protected by copyright but are freely available for you to use, provided you cite the reference in any questionnaires or publications.

### 4.3.6 Suggested Citation

O'Connor AM. User Manual - Decisional Conflict Scale (10 item question format) [document on the Internet]. Ottawa: Ottawa Hospital Research Institute; © 1993 [updated 2010; cited YYYY MM DD]. 16 p. Available from http://decisionaid.ohri.ca/docs/develop/User_Manuals/UM_Decisional_Conflict.pdf .

## 4.4 The SURE test version for clinical practice: 4 items 2 response categories

This version is recommended for use in everyday clinical practice.
Note: We always precede the DCS with an option preference question, which is not included in scoring. [See item 'A' below].

### 4.4.1 Scale

A. Which [insert treatment/screening] option do you prefer? Please check ☑ one.

a. ☐ **[Option 1]**
b. ☐ **[Option 2]**
c. ☐ **[Option 3]**
d. ☐ Unsure

| | | **Yes** [1] | **No** [0] |
|---|---|---|---|
| **S**ure of myself | Do you feel SURE about the best choice for you? | ☐ | ☐ |
| **U**nderstanding information | Do you know the benefits and risks of each option? | ☐ | ☐ |
| **R**isk-benefit ratio | Are you clear about which benefits and risks matter most to you? | ☐ | ☐ |
| **E**ncouragement | Do you have enough support and advice to make a choice? | ☐ | ☐ |

The SURE Test © O'Connor and Légaré, 2008.

| | | **Oui** [1] | **Non** [0] |
|---|---|---|---|
| **S**ûr | Êtes-vous certain de ce qui constitue le meilleur choix pour vous? | ☐ | ☐ |
| **U**tilité de l'information | Est-ce que vous connaissez les bénéfices et risques de chacune des options? | ☐ | ☐ |
| **R**isques-bénéfices à balancer | Avez-vous le sentiment de savoir ce qui est le plus important pour vous à l'égard des risques et bénéfices? | ☐ | ☐ |
| **E**ncouragement | Avez-vous suffisamment de soutien afin de faire votre choix? | ☐ | ☐ |

The SURE Test © O'Connor and Légaré, 2008.

### 4.4.2 Scoring and Interpretation

Items are given a score value of:
0 = 'no'; 1 = 'yes'.

TOTAL SCORE (UNCERTAINTY)
Can only be calculated if all items are answered.
The 4 items are summed.
Scores range from 0 [extremely high decisional conflict] to 4 [no decisional conflict].
A score of ≤ 3 indicates decisional conflict

### 4.4.3 Psychometric Properties

**In English:** The scale has been tested with 1474 patients referred to watch condition-specific video decision aids as part of their standard process of care. Alpha coefficient was 0.86. The scale is responsive to change from baseline (time 1) to the time following use of a video decision aid (T2) and counseling about options (T3) [22].

**In French:** The SURE test was completed by 123 French-speaking pregnant women registered at family medicine clinics in Quebec City [23].

| SURE score | N (%) | Proportion who had made choices | Proportion who had not made choices |
|---|---|---|---|
| 4 | 981 (67%) | 96 % | 4% |
| 3 | 272 (18%) | 66% | 34% |
| 2 | 147 (10%) | 59% | 41% |
| 1 | 54 (4%) | 54% | 46% |
| 0 | 20 (1%) | 65% | 35% |

Table 1. the SURE scores of patients in the treatment-option group (N=1474) and the proportion of them who had not made treatment choices.

**Reliability of the SURE**

The internal reliability of SURE was moderate (Cronbach α was 0.54 in French-speaking pregnant women and 0.65 in English-speaking treatment-option patients).

In the group of pregnant women, removing 1 item (ie, support) produced a higher value (Cronbach α = 0.61).

In the group of treatment-option patients, all item-to-item correlations were positive and ranged from 0.46 to 0.71.

**Construct validation**

In the group of French-speaking pregnant women, the SURE score correlated negatively with the DCS score (r=-0.46; P < .0001). In the English-speaking treatment-option group, patients who had not made choices about treatment (n=225) had lower mean (SD) SURE scores than those who had (n=1249) (2.6 (1.0) vs 3.6 (0.8), respectively; P < .0001) (See table 1).

A factorial analysis of the SURE test in the group of pregnant women indicated that 2 factors accounted for 72% of the variance. Three items (ie, knowledge, value, and certainty) loaded under 1 factor. The other item (ie, support) loaded under the second factor. In the treatment-option group, 1 factor accounted for 49% of the variance.

| Patient group and Conditions | N | Yes Responses, % | | | |
|---|---|---|---|---|---|
| | | Sure of myself | Understand information | Risk-benefit ratio | Ecouragement |
| French-speaking pregnant women | | | | | |
| • Prenatal screening | 123 | 87 | 98 | 94 | 98 |
| English-speaking treatment-option patients, Total N=1474 | | | | | |
| • Hip osteoarthritis | 160 | 80 | 99 | 95 | 94 |
| • Knee osteoarthritis | 292 | 75 | 98 | 95 | 90 |
| • Herniated disk | 177 | 76 | 99 | 93 | 93 |

| Patient group and Conditions | N | Yes Responses, % | | | |
|---|---|---|---|---|---|
| | | **Sure of myself** | **Understand information** | **Risk-benefit ratio** | **Ecouragement** |
| • Spinal stenosis | 295 | 71 | 95 | 90 | 84 |
| • Chronic back pain | 171 | 75 | 89 | 89 | 80 |
| • Prostate cancer | 204 | 59 | 96 | 90 | 77 |
| • Breast cancer reconstruction | 86 | 74 | 97 | 93 | 86 |
| • Early-stage breast cancer surgery | 89 | 60 | 96 | 87 | 84 |

Table 2. Percentage of participants responding yes to each of the 4-item SURE questions, by conditions

| Participant group | N | Correlation with Total | | | |
|---|---|---|---|---|---|
| | | **Sure of myself** | **Understand information** | **Risk-benefit ratio** | **Encouragement** |
| French-speaking pregnant women | 123 | 0.47 | 0.32 | 0.59 | 0.07 |
| English-speaking treatment-option patients | 1474 | 0.46 | 0.33 | 0.45 | 0.49 |

Table 3. Item-to-total Pearson correlation coefficient results for the 2 participant groups

### 4.4.4 Applications using this Tool

The tool has been used in 2 studies. Further research should assess the performance of the SURE test with a more diverse group of patients.

### 4.4.5 Other Languages

This tool is available in English and in French.

### 4.4.6 Availability

**You may use any of these scales at no cost without permission.**

These tools are protected by copyright but are freely available for you to use, as long as you cite the reference in any questionnaires or publications.

### 4.4.7 Suggested Citations

For the SURE tool:
Légaré F, Kearing S, Clay K, Gagnon S, D’Amours D, Rousseau M, O’Connor AM. Are you SURE? Assessing patient decisional conflict with a 4-item screening test. Can Fam Physician 2010; 56:e308-314.

For this User Manual:
O'Connor AM. User Manual - Decisional Conflict Scale [document on the Internet]. Ottawa: Ottawa Hospital Research Institute; © 1993 [updated 2010; cited YYYY MM DD]. 16 p. Available from http://decisionaid.ohri.ca/docs/develop/User_Manuals/UM_Decisional_Conflict.pdf .

## *5. Annotated References*

1. O'Connor AM. Validation of a decisional conflict scale. Med Dec Making 1995; 15(1): 25-30. The classic psychometric paper.
2. Brehaut JC, O'Connor AM, Wood TJ, Hack TF, Siminoff L, Gordon E, Feldman-Stewart D. Validation of a decision regret scale. Medical Decision Making 2003; 23:000-000. Shows correlation between DCS and regret.
3. O'Connor AM, Stacey D, Rover D, Holmes-Rovner M, Tetroe J, Llewellyn-Thomas H, Entwistle V, Rostom A, Fiset V, Barry M, Jones J. The Cochrane Databases of Systematic Reviews. The Cochrane Library, Volume (1) 2003. There are several studies that used DCS…note the smaller effect size when a simpler decision aid is compared to decision aid than when decision aid compared to usual care.
4. O'Connor AM, Fiset V, DeGrasse C, Graham ID, Evans W, Stacey D, Laupacis A, Tugwell P. Decision Aids for Patients Considering Options Affecting Cancer Outcomes: Evidence of Efficacy and Policy Implications. Journal of the National Cancer Institute Monographs, No. 25, 1999. Several before/after studies cited here.
5. O'Connor AM, Decisional conflict (Specify). Nursing diagnosis and intervention, 3rd ed., 486-496. Construct defined in first (1989) and subsequent editions.
6. Patient Decision Aid Research Group url for accessing evaluation measure information http://decisionaid.ohri.ca/eval.html.
7. O'Connor AM, Tugwell P, Wells GA, Elmslie T, Jolly E, Hollingworth G, McPherson R, Drake E, Hopman W, Mackenzie T. Randomized Trial of a Portable, Self-administered Decision Aid for Postmenopausal Women Considering Long-term Preventive Hormone Therapy. Medical Decision Making 1998; 18:295-303. USED TRADITIONAL DCS.
8. Laupacis A, O'Connor AM, Drake ER, Rubens FD, Robblee JA, Grant FC, Wells PS. A decision aid for autologous pre-donation in cardiac surgery – a randomized trial. Patient Education and Counselling (in press). USED TRADITIONAL DCS.
9. Man-Son-Hing M, Laupacis A, O'Connor AM, Biggs J, Drake E, Yetisir E, Hart RG. A Patient Decision Aid Regarding Antithrombotic Therapy for Stroke Prevention in Atrial Fibrillation, A Randomized Controlled Trial. JAMA. 1999; 282: 737-743. USED TRADITIONAL DCS
10. Dodin S, Legare F, Daudelin G, Tetroe J, O'Connor AM. Prise de décision en matière d'hormonothérapie de replacement, Essai clinique randomise. Canadian Family Physician 2001; 47: 1586-1593. USED FRENCH TRADITIONAL DCS
11. Feldman Stewart D, Brundage MD, Manen LV. A decision aid for men with early stage prostate cancer: theoretical basis and a test by surrogate patients. Health Expectations 2001; 4: 221-234. USED TRADITIONAL DCS
12. Siminoff LA, Ravdin P, Colabianchi N, Saunders Sturm CM. Doctor-patient communication patterns in breast cancer adjuvant therapy discussions. Health Expectations 2000; 3: 26-36. USED TRADITIONAL DCS
13. Morgan MWA randomized trial of the ischemic heart disease shared decision making program: an evaluation of a decision aid. University of Toronto; 1997. USED TRADITIONAL DCS
14. Murray E, Davis H, Tai SS, Coulter A, Gray A, Haines A. Randomised controlled trial of an interactive multimedia decision aid on hormone replacement therapy in primary care. BMJ 2001; 323: 490-3. USED TRADITIONAL DCS

15. Murray E, Davis H, Tai SS, Coulter A, Gray A, Haines A. Randomised controlled trial of an interactive multimedia decision aid on benign prostatic hypertrophy in primary care. BMJ 2001; 323: 493-6. USED TRADITIONAL DCS
16. Janis IL, Mann L. Decision Making. New York: The Free Press, 1977. Psychological basis for defining the construct.
17. North American Nursing Diagnosis Association. 10th Conference on Classification of Nursing Diagnoses. San Diego: California, 1992. Definition from a standard taxonomy of nursing problems.
18. Sun, Qiao. Predicting Downstream Effects of High Decisional Conflict: Meta-analyses of the Decisional Conflict Scale Unpublished MSc thesis. University of Ottawa, 2005. Demonstrates the predictive validity of the DCS on option knowledge, decision delay, discontinuance, and regret.
19. Gattelari & Ward J Med Screen 2004;11:165-169. Shows independent effect of decisional conflict on tendency to blame doctor for bad outcomes if they forgo PSA testing for prostate cancer.
20. Urrutia M, Campos S, O'Connor A. Validación de una versión en español de la Escala de Conflicto Decisional. Revita Médica de Chile 2008; 136:1439-1447.
21. LeBlanc A, Kenny DA, O'Connor AM, Légaré F. Decisional conflict in patients and their physicians: a dyadic approach to shared decision making. Med Decis Making. 2009 Jan-Feb;29(1):61-8. Epub 2009 Feb 4.
22. Légaré F, Kearing S, Clay K, Gagnon S, D'Amours D, Rousseau M, O'Connor AM. Are you SURE? Assessing patient decisional conflict with a 4-item screening test. Can Fam Physician 2010; 56:e308-314.
23. Gagnon S, Labrecque M, Njoya M, Rousseau F, St-Jacques S, Légaré F. How much do family physicians involve pregnant women in decisions about prenatal screening for Down syndrome? Prenat Diagn. 2010 Feb; 30(2): 115-121.
