# Supplementary material for: The effectiveness of decision aids for pregnancy related decision-making in women with pre-pregnancy morbidity; systematic review and meta-analysis
Source: BMC Pregnancy Childbirth. 2022 Jan 29;22:81. doi: 10.1186/s12884-022-04402-x (PMC8801107; doi:10.1186/s12884-022-04402-x)
Supplement: Supplementary file 3 — Additional file 3. [file 12884_2022_4402_MOESM3_ESM.docx]

Supplementary file x: Egger’s test for publication bias analysed by A. all ten studies B. pre-defined sub-groups pre-existing medical conditions and pre-existing surgical conditions

A.

B.
